# Efficacy and Safety of Initial 5 Years of Adjuvant Endocrine Therapy in Postmenopausal Hormone Receptor-Positive Breast Cancer: A Systematic Review and Network Meta-Analysis

**DOI:** 10.3389/fphar.2022.886954

**Published:** 2022-05-30

**Authors:** Hao Liao, Wendi Pei, Jianxin Zhong, Bin Shao, Xiaoran Liu, Yaxin Liu, Jiayang Zhang, Hope S. Rugo, Huiping Li

**Affiliations:** ^1^ Key Laboratory of Carcinogenesis and Translational Research (Ministry of Education/Beijing), Department of Breast Oncology, Peking University Cancer Hospital and Institute, Beijing, China; ^2^ Center for Reproductive Medicine, Beijing Key Laboratory of Reproductive Endocrinology and Assisted Reproductive Technology and Key Laboratory of Assisted Reproduction, Department of Obstetrics and Gynecology, Ministry of Education, Peking University Third Hospital, Beijing, China; ^3^ University of California, San Francisco Comprehensive Cancer Center, San Francisco, CA, United States

**Keywords:** adjuvant endocrine therapy, early breast cancer, network meta-analysis, tamoxifen, aromatase inhibitor

## Abstract

**Purpose:** To identify the optimal initial 5 years of adjuvant endocrine therapy for hormone receptor-positive postmenopausal early breast cancer (EBC) patients.

**Methods:** We conducted a systematic search of the PubMed, Web of Science, and EMBASE to obtain relevant studies published between January 2000 and January 2022. Randomized clinical trials assessing the efficacy and safety of initial 5 years of adjuvant endocrine therapy were included. The primary outcomes were disease-free survival and overall survival and the secondary outcome was severe adverse effects (SAEs). A Bayesian network meta-analysis was carried out to indirectly compare all regimens and the value of surface under the cumulative ranking curve (SUCRA) was used to obtain rankings.

**Results:** Eleven studies with 49,987 subjects were included. For DFS, exemestane (EXE) [hazard ratio (HR) 0.91, 95% confidence interval (95%CI) 0.87–0.96], anastrozole (ANA) (0.94, 0.90–0.97), letrozole (LET) (0.93, 0.89–0.97), tamoxifen (TAM) followed by EXE (0.91, 0.87–0.96), and TAM followed by ANA (0.92, 0.87–0.98) were more favorable than TAM, with TAM followed by EXE ranking as the first of SUCRA. For OS, only TAM followed by ANA showed significant superiority than TAM (HR 0.91, 95%CI 0.86–0.97) and ranked as the first of SUCRA. For SAEs, EXE (HR 1.72, 95%CI 1.04–2.98), ANA (1.58, 1.03–2.43), and LET (1.63, 1.02–2.57) showed greater associations with bone fracture than TAM. However, no significant difference in the incidences of cardiac events, thromboembolic events, and cerebrovascular events was found among all comparisons.

**Conclusion:** The sequential use of aromatase inhibitors, which has the best curative effects and relatively mild side effects, may be the optimal treatment mode for hormone receptor-positive postmenopausal EBC patients. In addition, the three kinds of aromatase inhibitors achieved roughly equal efficacy, but caused different types of SAEs.

**Systematic Review Registration:** [website], identifier [registration number].

## Introduction

Female breast cancer represents the most commonly diagnosed malignancy all over the world, with an estimated 2.3 million new cancer cases diagnosed in 2020 (Sung et al., 2021). Hormone receptor-positive (estrogen receptor positive and/or progesterone receptor positive) breast cancer is the most common type of breast cancer, accounting for nearly 80% of all new cases (DeSantis et al., 2019). Endocrine therapy is available for patients with hormone receptor-positive breast cancer and has greatly improved the clinical outcomes. For postmenopausal women with hormone receptor-positive early breast cancer (EBC), tamoxifen (TAM) had been established as the gold standard of adjuvant endocrine therapy for nearly 30 years, which acted as an antagonist of estrogen by saturating the estrogen receptor (Baum, 1983; Osborne, 1998). After 5 years of treatment with TAM, the risk of breast cancer recurrence and the risk of death were reduced by 47 and 26%, respectively (Clarke et al., 1998). Nevertheless, close to half of patients eventually acquired resistance to TAM and relapsed. Moreover, lengthy use of TAM was associated with increased incidences of severe adverse events (SAEs), including gynecological complications and thromboembolic events (Abe et al., 2005).

The third-generation aromatase inhibitors (AIs) came out in the middle of 1990s, including two nonsteroidal agents (anastrozole, ANA and letrozole, LET) and one steroidal agent (exemestane, EXE). In contrast to the receptor binding capacity of TAM, AIs reduced the production of estrogen in tissue and plasma by preventing the conversion from androgen to estrogen in postmenopausal women (Smith and Dowsett, 2003). Oral administration of AIs could result in an inhibition rate of aromatase activity of more than 99% (Lønning, 2004). Based on this rationale, AIs were expected to induce greater efficacy for postmenopausal hormone receptor-positive patients than TAM. Indeed, in the ATAC trial, 5 years of ANA significantly prolonged the disease-free survival (DFS) of postmenopausal patients with hormone receptor-positive EBC than 5 years of TAM [hazard ratio (HR) 0.83, 95% confidence interval (95%CI) 0.71–0.96, *p* = 0.013], and this improvement was still significant after a median follow-up of 120 months (HR 0.86, 95%CI 0.78–0.95, *p* = 0.003) (Baum et al., 2002; Cuzick et al., 2010). The following BIG 1-98 study also revealed an 18% reduction in the risk of an event ending a period of DFS in patients receiving LET than TAM (HR 0.81, 95%CI 0.70–0.93, *p* = 0.003) (Thurlimann et al., 2005). With these results, it was recommended to incorporate AIs into the initial 5 years of endocrine therapy (Burstein et al., 2010).

Besides the upfront use of AIs, several large randomized controlled trials (RCTs) have explored the efficacy of switching to an AI after 2–3 years of TAM (Coombes et al., 2007; Kaufmann et al., 2007; Dubsky et al., 2012; Boccardo et al., 2013). Both the IES (TAM followed by EXE vs. TAM, HR 0.76, 95%CI 0.66–0.88, *p* = 0.0001) and the ITA trial (TAM followed by ANA vs. TAM, HR 0.71, 95%CI 0.52–0.97, *p* = 0.005) revealed significant improvements in DFS of switch strategy compared with TAM alone (Coombes et al., 2007; Boccardo et al., 2013). The combined results of the ABCSG-8 study and the ARNO trial 95 also indicated an improvement of 40% in DFS of switching from TAM to ANA (Jakesz et al., 2005; Kaufmann et al., 2007; Dubsky et al., 2012). As a result, the sequential use of TAM and an AI was another practical option for postmenopausal hormone receptor-positive EBC, especially for those who would early develop resistance to TAM or had relatively higher risk of endometrial cancer and deep venous thrombosis.

However, no significance in DFS was observed between 5 years of EXE and TAM followed by EXE for 5 years (HR 0.96, 95%CI 0.88–1.05, *p* = 0.39) (Derks et al., 2017). Furthermore, the head-to-head comparisons between two individual AIs could not conclude which AI was relatively better (Goss et al., 2013; Smith et al., 2017). Apparently, there was an unmet need to identify the potentially best regimen of initial adjuvant endocrine therapy. Therefore, in this network meta-analysis, we synthesized the latest evidence to indirectly compare the efficacy and safety among different 5 years of regimens of initial adjuvant endocrine therapy.

## Methods

This study was carried out in accordance with the Cochrane Handbook (Cumpston et al., 2019) and reported based on the Preferred Reporting Items for Systematic Reviews and Meta-analysis (PRISMA) statement (Hutton et al., 2015).

### Search Strategy

We searched bibliographic databases in PubMed, EMBASE, and Web of Science to obtain relevant studies published between January 2000 and January 2022. MeSH terms and free text were combined to search for concepts such as “Breast Neoplasms”, “Receptors, Estrogen”, “positive” and “endocrine therapy”. The full search strategy and detailed sources of records were available in Supplementary Appendix S1. In addition, we manually searched the reference lists of included studies to identify other potentially eligible papers.

### Study Selection

Studies meeting the following criteria were included in the analysis: 1) RCTs; 2) postmenopausal adult female patients (≥18 years old) with histologically confirmed invasive breast cancer; 3) positive for estrogen receptors and/or progesterone receptors (≥1% of tumor nuclei positive in immunohistochemistry) (Allison et al., 2020); 4) local treatment with curative intent including surgery and radiation has been completed; 5) initial endocrine therapy with 5 years of regimens of TAM, AIs, or sequential TAM and an AI; 6) measurements of DFS, overall survival (OS), and SAEs; and 7) written in English. In particular, if there were multiple publications for one trial, only the most recently reported endpoints would be included. We excluded studies if they met one of the following terms: 1) non-RCT studies; 2) non-English publications; 3) patients in neoadjuvant or advanced settings; and 4) lacking control or inappropriate control group.

### Data Extraction and Risk of Bias Assessment

Two independent reviewers (HaL and WP) extracted the data from included studies according to a pre-specified protocol. The following study characteristics were collected: study name, publication year, study design, patient population, treatment strategy, sample size, median follow-up, and main outcomes. Discrepancies were settled by discussion. The primary outcomes of this study included DFS and OS. The secondary outcome was SAEs. DFS was defined as the time from randomization to recurrence of tumor or death. OS was defined as the time between diagnosis and death for any cause. SAEs included four life-threatening events: bone fracture, cardiac events, thromboembolic events, and cerebrovascular events.

The same two reviewers independently assessed the risk of bias for each study using the Cochrane Risk of Bias tool and evaluated them as high, low, or unclear risk (Higgins et al., 2011). Differences in opinion between the two reviewers in particular studies were resolved by discussion.

### Statistical Methodology

At first, we conducted traditional meta-analysis using Review Manager (version 5.3.5) to compare the efficacy of TAM, AIs, and TAM followed by an AI with a fixed-effects model. HRs with 95%CIs were calculated using the inverse variance method for time-to-event data. Pooled results were presented through forest plots. Both the Cochrane *Q* test and Higgins *I* ^2^ index were used to assess heterogeneity across studies, with a *p*-value of <0.1 and an *I* ^2^ value of >50% indicating significant heterogeneity (Lau et al., 1997). The potential publication bias was assessed by performing Egger’s and Begg’s test. Then, we employed a network meta-analysis to synthesize the therapeutic effects and safety of different regimens. We directly extracted the data from included studies to calculate the log HR for time-to-event data (DFS and OS) and the log odds ratio (OR) for dichotomous variable (rate of SAEs). For all three outcomes, the efficacy/safety of one regimen was superior than the other one if the corresponding HR/OR value was less than 1.

This network meta-analysis was conducted in the OpenBUGS 3.2.3 (www.openbugs.net) and GeMTC 0.14.3 (http://drugis.org/software/addis1/gemtc) for survival data (DFS and OS) and SAEs, respectively. A Bayesian fixed-effects model via Markov Chain Monte Carlo modeling (Stewart et al., 2014) was constructed to synthesize direct and indirect comparisons with the following parameters: number of chains, three; initial value, 0.5; number of simulation iterations, 30,000; number of adaptations, 3,000; and thinning factor, 10. In order to rank all regimens, we calculated the surface under the cumulative ranking curve (SUCRA). One regimen would be the best if its SUCRA value was 1, whereas one regimen would be the worst if its SUCRA value was 0 (Salanti et al., 2011). The model inconsistency in OpenBUGS was evaluated by Deviance Information Criterion (DIC), while random effects standard deviation and the value of inconsistency factor were used for the inconsistency assessment in GeMTC. Low DIC value, inconsistency factor approach to 0, and roughly equal random effects standard deviation between the consistency model and the inconsistency model indicated that the model was consistent (Dahabreh et al., 2012; Dias et al., 2013).

## Results

### Search Results and Study Characteristics

**FIGURE 1:**
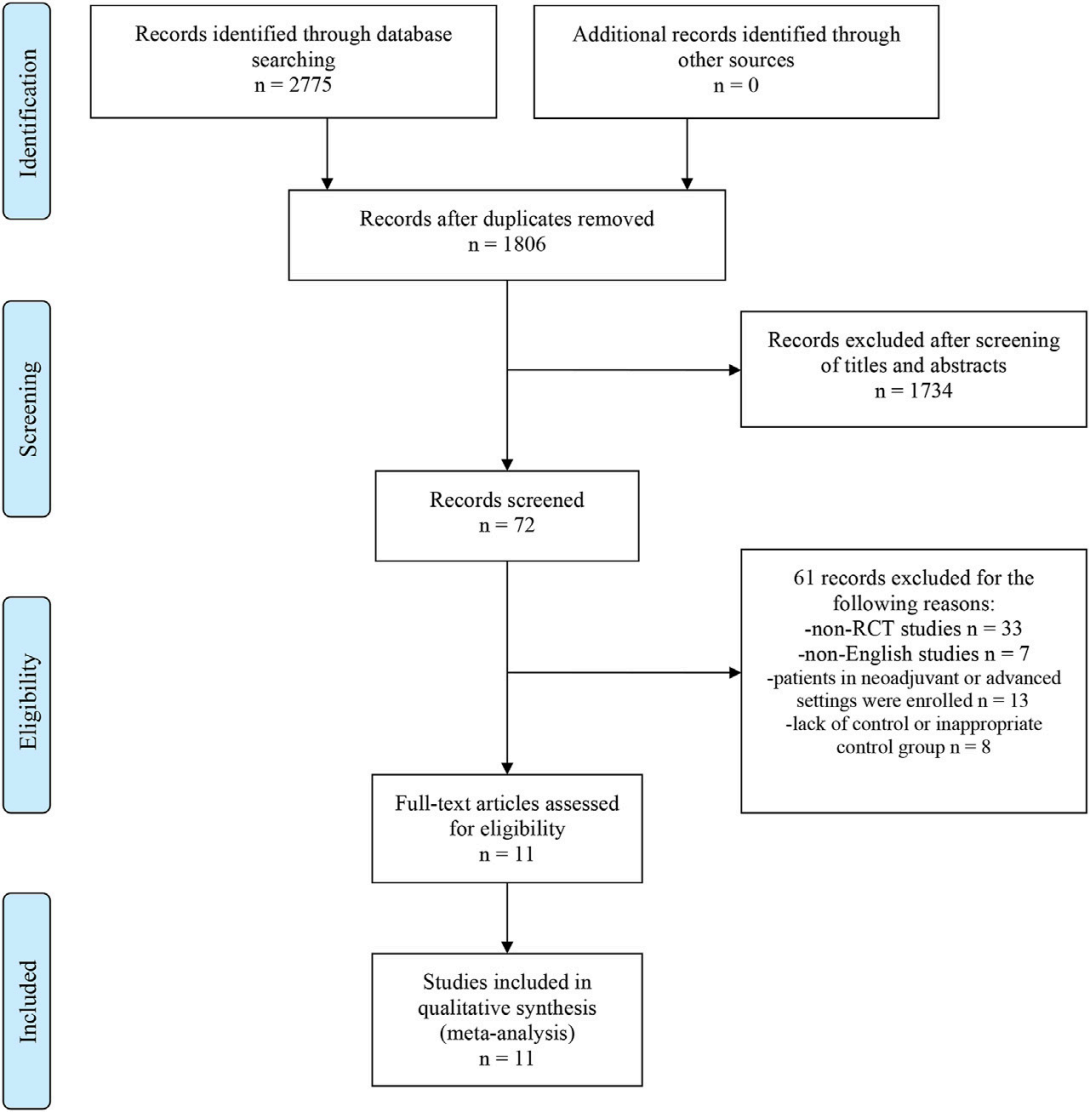
Flow Diagram of study selection process. Abbreviations: PRISMA, Preferred Reporting Items for Systematic Reviews and Meta-Analysis; RCT, randomized controlled trial.

The flow diagram of detailed screening process was presented in Figure 1. A total of 2775 records were identified by searching electronic databases. After removing duplicates, we excluded 1734 irrelevant records by screening titles and abstracts. Through reviewing full-text articles, we excluded 61 records with the following reasons (Sung et al., 2021): non-RCT studies, *n* = 33 (DeSantis et al., 2019); non-English studies, n = 7 (Baum, 1983); patients in neoadjuvant or advanced settings were enrolled, *n* = 13; and (Osborne, 1998) lack of control or inappropriate control group, *n* = 8. Eventually, 11 studies with 49,987 patients were eligible for this network meta-analysis (Coombes et al., 2007; Kaufmann et al., 2007; Mouridsen et al., 2009; Cuzick et al., 2010; Dubsky et al., 2012; Boccardo et al., 2013; Goss et al., 2013; Aihara et al., 2014; Derks et al., 2017; Smith et al., 2017; De Placido et al., 2018). No extra studies were obtained by searching the reference lists of these articles. The study characteristics of included studies were shown in Table 1. All of included studies were phase III RCTs, of which eight were open-label and three were double-blind. In terms of the treatment strategy, two, five, two, and two trials compared the efficacy and safety between 5 years of an AI and 5 years of TAM, TAM followed by an AI for 5 and 5 years of TAM, TAM followed by an AI for 5 and 5 years of an AI, and 5 years of an AI and 5 years of another AI. The median follow-up ranged from 2.5 to 10 years (eight studies, ≥5 years; three studies, <5 years). All articles were published after 2000 years and reported the latest data of their own. In particular, for studies enrolling all kinds of subtypes of EBC, we only analyzed the data from hormone receptor-positive subset. The original data of DFS, OS, and SAEs were presented in Supplementary Appendix S2.

**TABLE 1:** Basic characteristics of included studies.

Study	Publication year	Design	Patient population	Treatment arms	Population(n) arm 1:arm 2	Median follow-up	Main outcomes
GIM3-FATA (De Placido et al., 2018)	2018	Phase III, open-label	Postmenopausal women with HR+ EBC	(Sung et al., 2021) 5 years of AIs (DeSantis et al., 2019) 2 years of TAM followed by 3 years of AIs (ANA, EXE, LET)	1850:1847	5 years	1, 2, 3
FACE (Smith et al., 2017)	2017	Phase III, open-label	Postmenopausal women with HR+ and node-positive stage IIA-IIIC EBC	(Sung et al., 2021) 5 years of LET (DeSantis et al., 2019) 5 years of ANA	2061:2075	5.4 years	1, 2, 3, 4, 8
TEAM (Derks et al., 2017)	2017	Phase III, open-label	Postmenopausal women with HR+ EBC	(Sung et al., 2021) 2.5–3.0 years of TAM followed by EXE for a total of 5 years (DeSantis et al., 2019) 5 years of EXE	4868:4898	9.8 years	1, 2, 3, 5
N-SAS BC03 (Mouridsen et al., 2009)	2014	Phase III, open-label	Postmenopausal women with HR+ EBC	(Sung et al., 2021) 1–4 years of TAM followed by ANA for a total of 5 years (DeSantis et al., 2019) 5 years of TAM	345:351	8.1 years	1, 3, 5
ITA (Coombes et al., 2007)	2013	Phase III, open-label	Postmenopausal women with HR+ and node-positive EBC	(Sung et al., 2021) TAM followed by ANA for 5 years (DeSantis et al., 2019) 5 years of TAM	223:225	10.7 years	1, 2, 3, 6
MA27 (Goss et al., 2013)	2013	Phase III, open-label	Postmenopausal women with HR+ EBC	(Sung et al., 2021) 5 years of EXE (DeSantis et al., 2019) 5 years of ANA	3789:3787	4.1 years	1, 2, 3, 4, 6, 7
ABCSG-8 (Dubsky et al., 2012)	2012	Phase III, open-label	Postmenopausal women with HR+ EBC	(Sung et al., 2021) 2 years of TAM followed by 3 years of ANA (DeSantis et al., 2019) 5 years of TAM	1865:1849	5 years	1, 2, 3, 5, 10
ATAC (Cuzick et al., 2010)	2010	Phase III, double-blind	Postmenopausal women with EBC	(Sung et al., 2021) 5 years of ANA (DeSantis et al., 2019) 5 years of TAM	3125:3116 (2618:2598 for HR+ patients)	10 years	1, 2, 3, 7, 8, 9, 11
BIG1-98 (Aihara et al., 2014)	2009	Phase III, double-blind	Postmenopausal women with HR+ EBC	(Sung et al., 2021) 5 years of LET (DeSantis et al., 2019) 5 years of TAM	4003:4007	6.3 years	1, 2, 3, 4
IES (Boccardo et al., 2013)	2007	Phase III, double-blind	Postmenopausal women with EBC	(Sung et al., 2021) 2–3 years of TAM followed by 2–3 years of EXE (DeSantis et al., 2019) 5 years of TAM	2352:2372	4.6 years	1, 2, 3, 7
ARNO95 (Kaufmann et al., 2007)	2007	Phase III, open-label	Postmenopausal women with HR+ EBC	(Sung et al., 2021) 2 years of TAM followed by 3 years of ANA (DeSantis et al., 2019) 5 years of TAM	489:490	2.5 years	1, 2, 3

Notes: *Main outcomes: 1, DFS; 2, OS; 3, safety; 4, distant DFS; 5, RFS; 6, EFS; 7, contralateral breast cancer; 8, time to distant recurrence; 9, time to recurrence; 10, distant RFS; 11, death with or without recurrence. Abbreviations: HR+, hormone receptor-positive; EBC, early breast cancer; AIs, aromatase inhibitors; TAM, tamoxifen; LET, letrozole; ANA, anastrozole; EXE, exemestane.

### Quality Assessment

**FIGURE 2:**
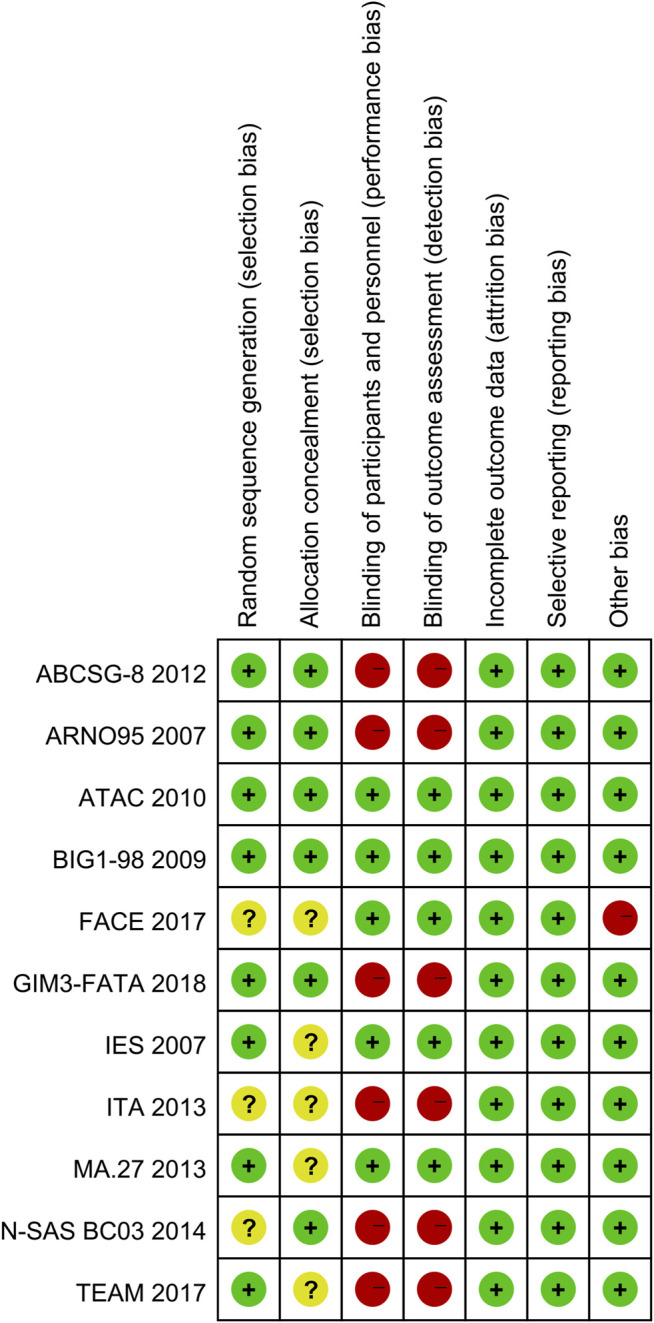
Risk of bias summary.

The risk of bias summary of included studies was as shown in Figure 2. Two studies were judged to be at low risk of bias (Cuzick et al., 2010; Aihara et al., 2014). Two studies were judged to be at unclear risk of bias by unclear methods of generating allocation sequences and setting allocation concealment (Boccardo et al., 2013; Goss et al., 2013). Seven studies were at high risk of bias (Coombes et al., 2007; Kaufmann et al., 2007; Mouridsen et al., 2009; Dubsky et al., 2012; Derks et al., 2017; Smith et al., 2017; De Placido et al., 2018). The major concern was that six studies with high risk of bias were open-label trials and no blinding of participants and personnel was performed. In addition, one study was judged to be at high risk in the domain of other bias because of early study termination (Smith et al., 2017).

### Traditional Meta-Analysis

**FIGURE 3:**
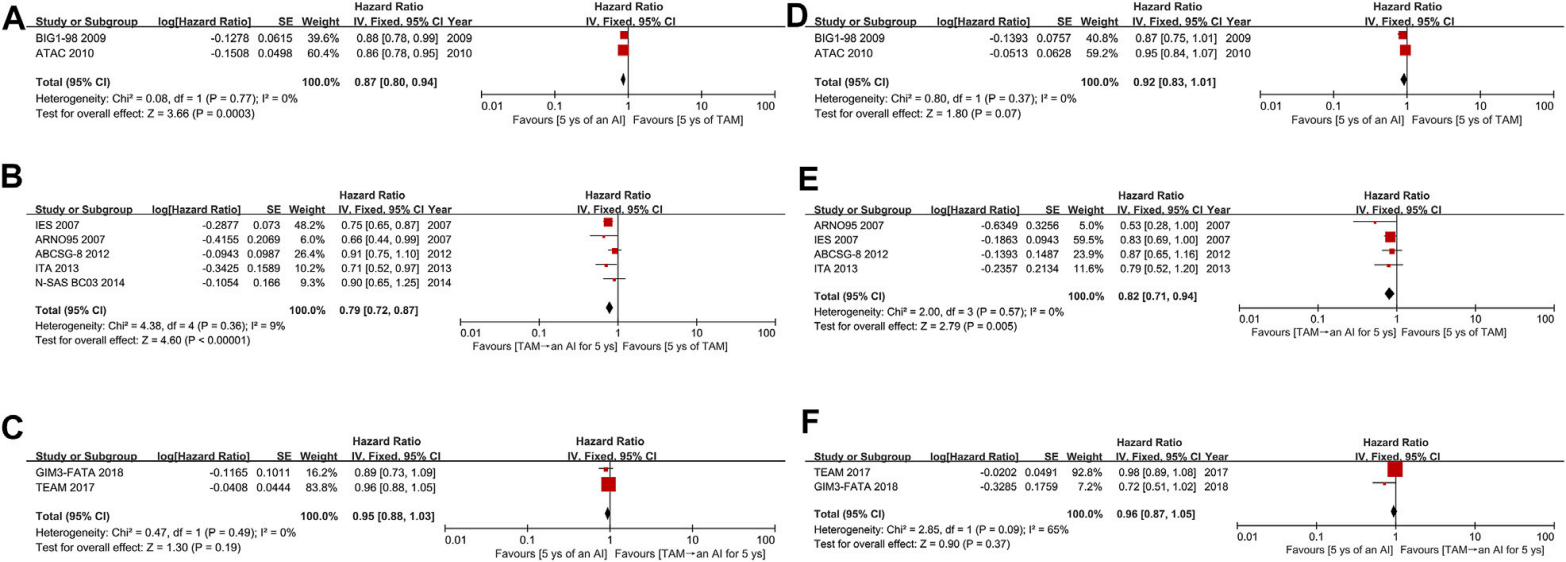
Forest plots for **(A–C)** DFS and **(D–F)** OS. Abbreviations: DFS, disease-free survival; OS, overall survival; AI, aromatase inhibitor; TAM, tamoxifen; ys, years.

All 11 studies reported survival outcomes in postmenopausal patients with HR+ EBC treated with initial 5 years of adjuvant endocrine therapy. Among them, nine (Coombes et al., 2007; Kaufmann et al., 2007; Mouridsen et al., 2009; Cuzick et al., 2010; Dubsky et al., 2012; Boccardo et al., 2013; Aihara et al., 2014; Derks et al., 2017; De Placido et al., 2018) and eight (Coombes et al., 2007; Kaufmann et al., 2007; Cuzick et al., 2010; Dubsky et al., 2012; Boccardo et al., 2013; Aihara et al., 2014; Derks et al., 2017; De Placido et al., 2018) studies could be used for the direct meta-analysis of DFS and OS, respectively. For DFS, both AIs (HR 0.87, 95%CI 0.80–0.94, *p* = 0.0003) and TAM followed by an AI (0.79, 0.72–0.87, *p* < 0.00001) were significantly better than TAM (Figures 3A,B), with low heterogeneity (*I* ^*2*^ = 0 and 9%, respectively). However, AIs did not significantly improve DFS than TAM followed by an AI (HR 0.95, 95%CI 0.88–1.03, *p* = 0.19) (Figure 3C). For OS, TAM followed by an AI was superior than TAM (HR 0.82, 95%CI 0.71–0.94, *p* = 0.005), with a low heterogeneity of *I* ^*2*^ = 0% (Figure 3E). Nevertheless, there was no significance between AIs and TAM (HR 0.92, 95%CI 0.83–1.01, *p* = 0.07), and AIs and TAM followed by an AI (0.96, 0.87–1.05, *p* = 0.37) (Figures 3D,F). The assessment of publication bias can only be conducted in DFS between TAM and TAM followed by an AI, and OS between TAM and TAM followed by an AI, as the other four comparisons only included two studies. The Egger’s (*p* = 0.892 and 0.249) and Begg’s test (*p* = 1 and 0.308) revealed no publication bias and the Funnel plots were shown in Supplementary Appendix S3.

### Network Meta-Analysis

**FIGURE 4:**
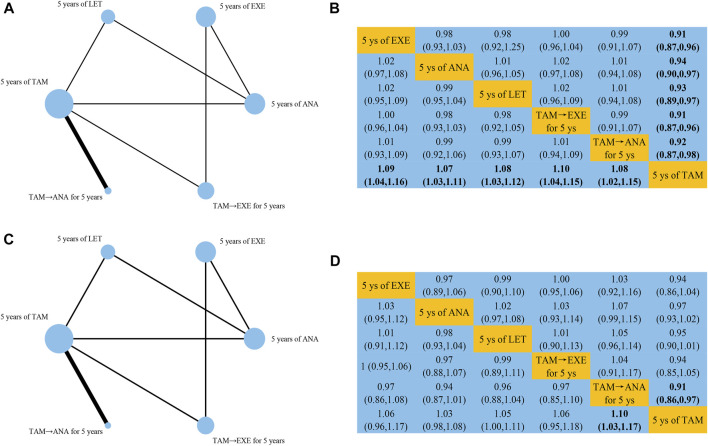
Network structure diagrams and league tables for **(A,B)** DFS and **(C,D)** OS. Abbreviations: EXE, exemestane; ANA, anastrozole; LET, letrozole.

Network structure diagrams for all analyses of DFS (Figure 4A), OS (Figure 4C), and SAEs (Supplementary Appendix S4) were plotted using the Network package in Stata 15.0 (Stata Corporation, College Station, TX, United States). The width of edge provided a measure of the number of direct comparisons between two regimens. The size of node was proportional to the number of randomized participants of each regimen. For example, both in DFS and OS, TAM ranked in the first among all regimens in the number of randomized participants and there were most direct comparisons between TAM and TAM followed by ANA.

### Disease-Free Survival

**FIGURE 5:**
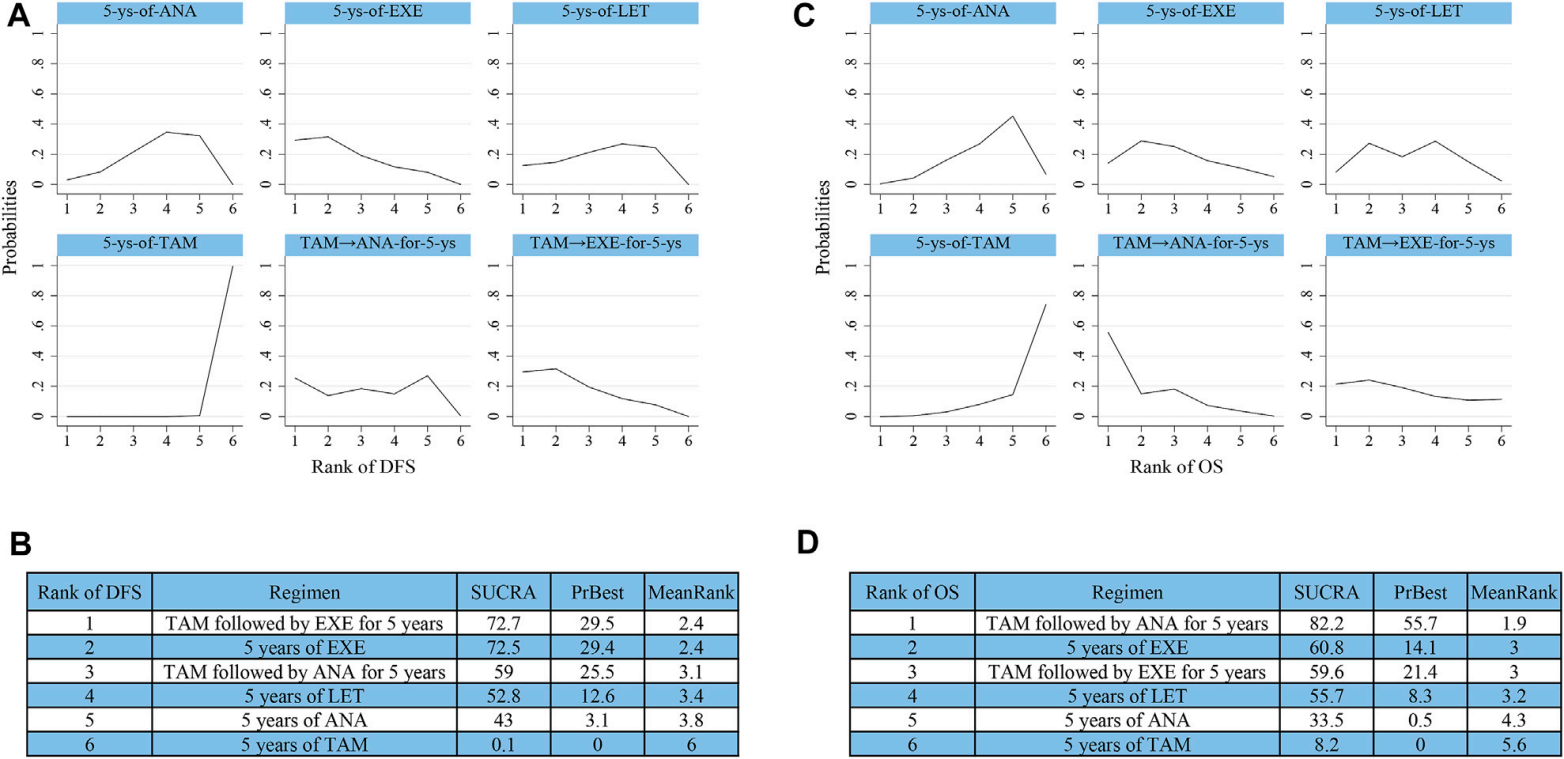
Ranking results of SUCRA for **(A,B)** DFS and **(C,D)** OS. Abbreviations: SUCRA, surface under the cumulative ranking curve.

10 studies reported data that could be used for the network meta-analysis of DFS (Coombes et al., 2007; Kaufmann et al., 2007; Mouridsen et al., 2009; Cuzick et al., 2010; Dubsky et al., 2012; Boccardo et al., 2013; Goss et al., 2013; Aihara et al., 2014; Derks et al., 2017; Smith et al., 2017). As shown in Figure 4B, EXE (HR 0.91, 95%CI 0.87–0.96), ANA (0.94, 0.90–0.97), LET (0.93, 0.89–0.97), TAM followed by EXE (0.91, 0.87–0.96), and TAM followed by ANA (0.92, 0.87–0.98) were significantly better than TAM. According to the cumulative SUCRA ranking curve (Figures 5A,B), TAM followed by EXE had the highest probability to be the best treatment (SUCRA 72.7, MeanRank 2.4), followed by EXE (72.5, 2.4), TAM followed by ANA (59, 3.1), LET (52.8, 3.4), ANA (43, 3.8), and TAM (0.1, 6).

### Overall Survival

Effects of initial 5 years of adjuvant endocrine therapy on OS were reported in nine studies (Coombes et al., 2007; Kaufmann et al., 2007; Cuzick et al., 2010; Dubsky et al., 2012; Boccardo et al., 2013; Goss et al., 2013; Aihara et al., 2014; Derks et al., 2017; Smith et al., 2017). The results of synthesized analysis indicated that only TAM followed by ANA showed significant superiority than TAM (HR 0.91, 95%CI 0.86–0.97) (Figure 4D). As shown in Figures 5C,D, TAM followed by ANA ranked as the best regimen (SUCRA 82.2, MeanRank 1.9), followed by EXE (60.8, 3), TAM followed by EXE (59.6, 3), LET (55.7, 3.2), ANA (33.5, 4.3), and TAM (8.2, 5.6). The DIC values of fixed-effects model were shown to be lower than that of random-effects model both in DFS (−29.98 vs. −29.11) and OS (−25.8 vs. −23.92). Thus, the fixed-effects model was chosen to reduce inconsistency in this network meta-analysis.

### Severe Adverse Events

Ten (Coombes et al., 2007; Kaufmann et al., 2007; Mouridsen et al., 2009; Cuzick et al., 2010; Dubsky et al., 2012; Boccardo et al., 2013; Goss et al., 2013; Aihara et al., 2014; Derks et al., 2017; Smith et al., 2017), nine (Coombes et al., 2007; Kaufmann et al., 2007; Mouridsen et al., 2009; Cuzick et al., 2010; Boccardo et al., 2013; Goss et al., 2013; Aihara et al., 2014; Derks et al., 2017; Smith et al., 2017), seven (Kaufmann et al., 2007; Mouridsen et al., 2009; Cuzick et al., 2010; Boccardo et al., 2013; Aihara et al., 2014; Derks et al., 2017; Smith et al., 2017), and six (Kaufmann et al., 2007; Cuzick et al., 2010; Goss et al., 2013; Aihara et al., 2014; Derks et al., 2017; Smith et al., 2017) of included studies reported the incidences of bone fracture, cardiac events, thromboembolic events, and cerebrovascular events in each group. The network plots for the four SAEs were shown in sequence in Supplementary Appendix S4. We noticed that EXE (HR 1.72, 95%CI 1.04–2.98), ANA (1.58, 1.03–2.43), and LET (1.63, 1.02–2.57) showed greater associations with bone fracture than TAM (Supplementary Appendix S5A). However, there was no significant difference in the incidences of cardiac events, thromboembolic events, and cerebrovascular events among all regimens (Supplementary Appendix S5B–D). According to the ranking results of SUCRA (Supplementary Appendix S6), EXE and LET could result in more bone fracture and cardiac events, respectively. TAM and TAM followed by ANA were potentially associated with higher risk of thromboembolic events and cerebrovascular events, respectively. The inconsistency factors from the analyses of all four SAEs were close to 0. Moreover, the random effects standard deviations between consistency model and inconsistency model were shown to be roughly equal (data not shown). In short, the analysis model applied in this network meta-analysis was consistent.

## Discussion

Hormone receptor-positive breast cancer patients tended to have a better prognosis compared with those with other subtypes due to the relatively low degree of malignancy and invasiveness. Stage I hormone receptor-positive breast cancers had a 5 years of breast cancer-specific survival of 99% and the median OS of stage IV diseases could reach 5 years (Waks and Winer, 2019). For postmenopausal women, the initial 5 years of adjuvant endocrine therapies primarily consisted of TAM, AIs, and TAM followed by an AI. Moreover, recent explorations of CDK4/6 inhibitors in adjuvant setting suggested that the addition of CDK4/6 inhibitors to standard endocrine therapy significantly improved the prognosis (Johnston et al., 2020; Gao et al., 2021). Therefore, in the era of CDK4/6 inhibitors, it seemed necessary to standardize the basic endocrine therapy and thereby reduce the discrepancy in outcomes resulted from drug differences. So far, the only two head-to-head studies (FACE and GIM3-FATA) comparing two AIs could not indicate which AI was better, though 5 years of an AI has been shown to be superior than TAM alone (Smith et al., 2017; De Placido et al., 2018). In order to identify the optimal initial endocrine therapy, we conducted this network meta-analysis using the results of the most recently updated studies.

Overall, this study included 11 phase III RCTs involving 49,987 postmenopausal women with hormone receptor-positive EBC, comparing the efficacy and safety of different initial 5 years of endocrine therapies. In traditional meta-analysis, we compared three treatment modes (TAM alone, the upfront use of AIs, and the sequential use of AIs). The results showed that both the upfront use of AIs and the sequential use of AIs were better than TAM alone in DFS. In terms of OS, only the sequential use of AIs was superior than TAM alone. These results were consistent with previous studies that regimens incorporating AIs could provide significant survival benefits (Thurlimann et al., 2005; Cuzick et al., 2010; Aromatase inhibitors versus tamoxifen in, 2015). Nevertheless, comparisons between the upfront use of AIs and the sequential use of AIs showed no significant difference in DFS and OS. This could be resulted from the differences in the nature of three AIs and make the following network analyses more interesting.

In the network model, a total of six regimens were included, which were EXE, ANA, LET, TAM followed by EXE, TAM followed by ANA, and TAM. For DFS, all five regimens that incorporated AIs were superior than TAM. This was consistent with the results of direct meta-analysis and the prevailing view that AIs were more active than TAM in treating hormone receptor-positive diseases in adjuvant setting (Thurlimann et al., 2005; Cuzick et al., 2010; Aromatase inhibitors versus tamoxifen in, 2015). The sequential use of EXE and the upfront use of EXE ranked as the top two of SUCRA for DFS. It was probably due to that EXE, as an irreversible suicide inhibitor, could maximize the benefits of DFS by its strongest inhibitory ability to estrogen (Boeddinghaus and Dowsett, 2001). Commonly, almost any form of anti-tumor therapies could cause side effects to some extent while eliminating tumor cells. In hormone receptor-positive diseases, the benefits in survival derived from endocrine therapy was achieved at the expense of damage to other aspects’ health (Coleman et al., 2007). In this study, only the sequential use of ANA was significantly better than TAM alone and ranked as the first of SUCRA for OS. We speculated that this was because ANA caused fewer SAEs such as bone fracture than EXE, which will be discussed in detail below.

As for SAEs, EXE, LET, and ANA had higher probabilities of causing bone fracture compared to TAM, ranking as the top three of SUCRA. These results could be explained by the major risk of AIs, namely accelerated bone resorption when estrogen conversion was almost completely inhibited (Chien and Goss, 2006). Although there was no significant difference in bone fracture rates among the three types of AIs, 5 years of EXE was the regimen most likely to cause bone fracture. This could be related to the stronger anti-estrogen effects of EXE compared to the other two AIs. However, the specific substudy on bone mineral density of MA.27 revealed no significant difference between hormone receptor-positive EBC patients receiving EXE and ANA regardless of the baseline bone mineral density T-score (Goss et al., 2014). Moreover, it was shown that EXE could exert mild androgenic effects as a steroidal agent and thereby reduce the extent of bone loss (Goss et al., 2004; Goss et al., 2007). These inconsistencies suggested the need for further research on the effects of AIs on bone-related events.

As for the analyses of the other three SAEs, there was no significant difference among the incidences of all regimens. As a selective estrogen receptor modulator, TAM’s roles varied among different organizations. For example, in breast tissue TAM acted as a potent estrogen antagonist by competitively binding to estrogen receptor, while in the heart TAM exerted estrogen-like protective actions (Christodoulakos et al., 2006). Moreover, it was found that TAM was associated with a protective effect to the arteries based on analyses of clinical data (Bradbury et al., 2005). In present study, indeed, TAM had the highest probability to be best regimen in terms of cardiac events. The cause of heart disease in LET monotherapy was unknown yet, which may be simply accidental or may be related to the lack of vascular protection of TAM (Thurlimann et al., 2005). For the incidence of thromboembolic events, TAM could be the worst regimen according to the results of SUCRA. This result was predictable to some extent because almost all included studies indicated significantly higher incidences of venous thrombosis in TAM alone than the other arm (ATAC, 3.5 vs. 2.1%; BIG1-98, 3.5 vs. 1.5%; IES, 2.3 vs. 1.2%; ARNO95, 1.3 vs. 0%). For the incidence of cerebrovascular events, TAM followed by ANA was shown to have the highest probability to be the worst regimen. This ranking result could be caused by a seemingly high ratio, since there were only three cases receiving the sequential use of ANA and one case receiving TAM alone who had cerebrovascular events in the entire analysis, respectively. Thus, this result should be interpreted with caution and further verified.

A previous network meta-analysis assessed the efficacy of adjuvant endocrine monotherapy (Yu et al., 2018). A total of 14 studies with 19,517 patients were included. The results indicated that LET and EXE might be the best agents for DFS and OS, respectively. This was consistent with one of our research conclusions that AIs were superior than TAM in terms of efficacy. However, they did not analyze the toxic effects due to incomplete and inconsistent data. Furthermore, there was no comparison about the sequential use of AIs in that study, which should be discussed emphatically since it has become an important part of adjuvant endocrine therapy. An earlier direct meta-analysis including seven RCTs compared the efficacy and safety among AIs, TAM, and the sequenced use of AIs (Rydén et al., 2016). Similar conclusion that AIs were better than TAM in efficacy was also obtained. Nevertheless, the improvement of the sequential use of AIs in OS was not significant compared with TAM alone. In our direct meta-analysis, the sequential use of AIs after TAM was shown to significantly improve OS, which may be resulted from a larger number of samples included in our study. This study also had several limitations. First, 10 out of 11 studies were carried out in western countries and regions, while only one study with 696 patients was conducted in Asia. This distribution bias of race may reduce the applicability of our findings. Second, seven out of 11 studies were evaluated as high risk of bias, which may lead to a decline in the quality of evidence. Third, some SAEs such as endometrial cancer and vaginal bleeding were not analyzed due to small numbers of studies.

## Conclusion

In summary, any regimens involving AIs improved the DFS of postmenopausal hormone receptor-positive EBC patients compared with TAM alone. Only the sequential use of AIs especially ANA was superior than TAM alone in OS. No significant difference of survival was found in the direct comparison between the upfront use and the sequential use of AIs, or in the indirect comparisons among different AIs. In terms of safety, the sequential use of AIs was generally associated with less SAEs than the upfront use of AIs, with the categories of SAEs varying among different regimens. From a long-term perspective, the sequential use of AIs may be the best treatment mode for postmenopausal hormone receptor-positive EBC patients. Therefore, when making clinical decisions, physicians need to balance short-term and long-term benefits, and select suitable agents according to patients’ clinical characteristics and potential risk of side effects.

## Data Availability

The original contributions presented in the study are included in the article/Supplementary Material, further inquiries can be directed to the corresponding author.

## Author Contricutions

HaL, WP, and JZ collected relevant literatures, extracted the data, and drafted the manuscript. BS, XL, YL, and JZ collected relevant literatures and conducted the statistical analysis. HR and HuL critically revised the manuscript for content. All authors read and approved the final manuscript.

## Abbreviations

AIs, aromatase inhibitors; ANA, anastrozole; CI, confidence interval; DFS, disease-free survival; EBC, early breast cancer; EXE, exemestane; DIC, Deviance Information Criterion; HR, hazard ratio; LET, letrozole; OR, odds ratio; OS, overall survival; PRISMA, Preferred Reporting Items for Systematic Reviews and Meta-analysis; RCTs, randomized controlled trials; SAEs, severe adverse effects; SUCRA, surface under the cumulative ranking curve; TAM, tamoxifen.
