# Supplementary material for: Efficacy and Safety of Initial 5 Years of Adjuvant Endocrine Therapy in Postmenopausal Hormone Receptor-Positive Breast Cancer: A Systematic Review and Network Meta-Analysis
Source: Front Pharmacol. 2022 May 30;13:886954. doi: 10.3389/fphar.2022.886954 (PMC9198062; doi:10.3389/fphar.2022.886954)
Supplement: Supplementary file 1 [file Image5.PDF]

5 ys of EXE	1.09 (0.70, 1.76)	1.05 (0.58, 1.99)	1.39 (0.86, 2.21)	1.36 (0.72, 2.88)	**1.72 (1.04, 2.98)**
0.92 (0.57, 1.43)	5 ys of ANA	0.97 (0.61, 1.56)	1.27 (0.73, 2.15)	1.26 (0.70, 2.42)	**1.58 (1.03, 2.43)**
0.95 (0.50, 1.72)	1.03 (0.64, 1.64)	5 ys of LET	1.31 (0.70, 2.36)	1.29 (0.71, 2.57)	**1.63 (1.02, 2.57)**
0.72 (0.45, 1.17)	0.79 (0.46, 1.36)	0.76 (0.42, 1.42)	TAM→EXE for 5 ys	0.99 (0.54, 1.99)	1.25 (0.79, 2.07)
0.73 (0.35, 1.39)	0.80 (0.41, 1.42)	0.77 (0.39, 1.40)	1.02 (0.50, 1.85)	TAM→ANA for 5 ys	1.26 (0.79, 1.90)
**0.58 (0.34, 0.96)**	**0.63 (0.41, 0.97)**	**0.61 (0.39, 0.98)**	0.80 (0.48, 1.26)	0.79 (0.53, 1.26)	5 ys of TAM

(a)

5 ys of EXE	1.27 (0.55, 2.97)	0.99 (0.35, 2.85)	1.25 (0.56, 2.68)	0.97 (0.29, 3.12)	1.35 (0.57, 3.32)
0.79 (0.34, 1.82)	5 ys of ANA	0.77 (0.35, 1.63)	0.98 (0.40, 2.28)	0.75 (0.26, 2.22)	1.05 (0.53, 2.10)
1.01 (0.35, 2.84)	1.30 (0.61, 2.82)	5 ys of LET	1.26 (0.47, 3.48)	0.99 (0.33, 2.98)	1.37 (0.66, 2.89)
0.80 (0.37, 1.78)	1.02 (0.44, 2.50)	0.80 (0.29, 2.12)	TAM→EXE for 5 ys	0.78 (0.26, 2.33)	1.08 (0.51, 2.36)
1.03 (0.32, 3.43)	1.32 (0.45, 3.87)	1.01 (0.34, 3.00)	1.28 (0.43, 3.87)	TAM→ANA for 5 ys	1.37 (0.63, 3.14)
0.74 (0.30, 1.77)	0.95 (0.48, 1.87)	0.73 (0.35, 1.50)	0.93 (0.42, 1.96)	0.73 (0.32, 1.59)	5 ys of TAM

(b)

5 ys of EXE	0.63 (0.01, 364.74)	0.69 (0.01, 303.67)	0.63 (0.03, 26.44)	2.42 (0.01, 799.94)	0.3 (0.00, 58.90)
1.60 (0.00, 183.84)	5 ys of ANA	1.07 (0.07, 11.80)	1.02 (0.01, 41.10)	3.11 (0.02, 378.64)	0.52 (0.03, 5.46)
1.45 (0.00, 198.84)	0.93 (0.08, 13.83)	5 ys of LET	0.94 (0.01, 41.82)	3.11 (0.02, 363.90)	0.48 (0.04, 6.07)
1.58 (0.04, 35.47)	0.98 (0.02, 100.54)	1.07 (0.02, 127.86)	TAM→EXE for 5 ys	3.50 (0.04, 582.32)	0.51 (0.03, 19.45)
0.41 (0.00, 112.88)	0.32 (0.00, 47.06)	0.32 (0.00, 61.57)	0.29 (0.00, 25.29)	TAM→ANA for 5 ys	0.16 (0.00, 8.04)
3.12 (0.02, 211.84)	1.94 (0.18, 32.66)	2.09 (0.16, 27.67)	1.96 (0.05, 34.24)	6.26 (0.12, 620.32)	5 ys of TAM

(c)

5 ys of EXE	0.82 (0.27, 2.59)	0.58 (0.13, 2.69)	1.45 (0.49, 4.39)	0.14 (0.00, 2.07)	0.46 (0.11, 1.89)
1.21 (0.39, 3.66)	5 ys of ANA	0.69 (0.28, 1.84)	1.72 (0.35, 7.88)	0.16 (0.01, 2.08)	0.56 (0.22, 1.40)
1.72 (0.37, 7.47)	1.44 (0.54, 3.56)	5 ys of LET	2.47 (0.39, 14.10)	0.23 (0.01, 2.79)	0.79 (0.30, 1.96)
0.69 (0.23, 2.03)	0.58 (0.13, 2.88)	0.40 (0.07, 2.54)	TAM→EXE for 5 ys	0.10 (0.00, 1.96)	0.32 (0.05, 1.95)
6.99 (0.48, 228.97)	6.08 (0.48, 154.87)	4.40 (0.36, 114.83)	10.43 (0.51, 353.90)	TAM→ANA for 5 ys	3.44 (0.34, 78.47)
2.16 (0.53, 8.94)	1.80 (0.71, 4.48)	1.26 (0.51, 3.34)	3.14 (0.51, 18.25)	0.29 (0.01, 2.98)	5 ys of TAM

(d)

Appendix 5. League tables of (a) bone fracture (b) cardiac events (c) thromboembolic events and (d) cerebrovascular events
Abbreviations: ANA, anastrozole; EXE, exemestane; LET, letrozole; TAM, tamoxifen; ys, years.
