# Supplementary material for: Efficacy and Safety of Initial 5 Years of Adjuvant Endocrine Therapy in Postmenopausal Hormone Receptor-Positive Breast Cancer: A Systematic Review and Network Meta-Analysis
Source: Front Pharmacol. 2022 May 30;13:886954. doi: 10.3389/fphar.2022.886954 (PMC9198062; doi:10.3389/fphar.2022.886954)
Supplement: Supplementary file 2 [file Image6.PDF]

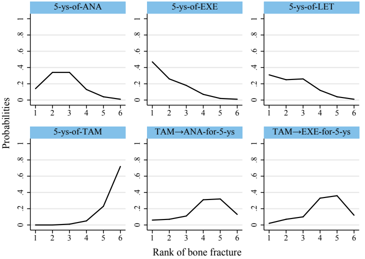

Rank of bone fracture	Regimen	SUCRA	PrBest	MeanRank
1	5 years of EXE	81.8	47	2
2	5 years of LET	72.2	31	2.3
3	5 years of ANA	67.6	14	2.6
4	TAM followed by ANA for 5 years	37	6	4.2
5	TAM followed by EXE for 5 years	34	2	4.3
6	5 years of TAM	7.2	0	5.7

(a)

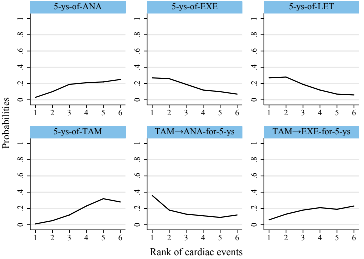

Rank of cardiac events	Regimen	SUCRA	PrBest	MeanRank
1	5 years of LET	67	27	2.6
2	TAM followed by ANA for 5 years	64.4	36	2.7
3	5 years of EXE	66	27	2.8
4	TAM followed by EXE for 5 years	39.4	6	4
5	5 years of ANA	35.2	3	4.2
6	5 years of TAM	27.8	1	4.7

(b)

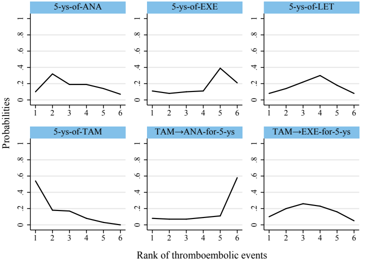

Rank of thromboembolic events	Regimen	SUCRA	PrBest	MeanRank
1	5 years of TAM	82.4	54	1.9
2	5 years of ANA	57.4	10	3.2
3	TAM followed by EXE for 5 years	54	10	3.3
4	5 years of LET	48	8	3.6
5	5 years of EXE	35.6	11	4.2
6	TAM followed by ANA for 5 years	23.6	8	4.8

(c)

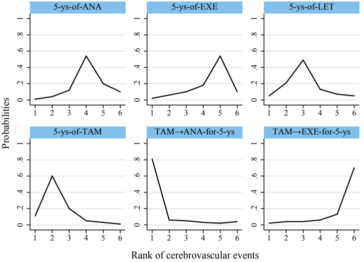

Rank of cerebrovascular events	Regimen	SUCRA	PrBest	MeanRank
1	TAM followed by ANA for 5 years	90.4	81	1.5
2	5 years of TAM	73.6	11	2.3
3	5 years of LET	57.8	5	3.1
4	5 years of ANA	37	1	4.2
5	5 years of EXE	30.8	2	4.5
6	TAM followed by EXE for 5 years	12.6	2	5.3

(d)

Appendix 6. Ranking results of SUCRA for (a) bone fracture (b) cardiac events (c) thromboembolic events and (d) cerebrovascular events

Abbreviations: ANA, anastrozole; EXE, exemestane; LET, letrozole; TAM, tamoxifen; ys, years.
