# Supplementary material for: Efficacy and Safety of Initial 5 Years of Adjuvant Endocrine Therapy in Postmenopausal Hormone Receptor-Positive Breast Cancer: A Systematic Review and Network Meta-Analysis
Source: Front Pharmacol. 2022 May 30;13:886954. doi: 10.3389/fphar.2022.886954 (PMC9198062; doi:10.3389/fphar.2022.886954)
Supplement: Supplementary file 3 [file Image4.PDF]

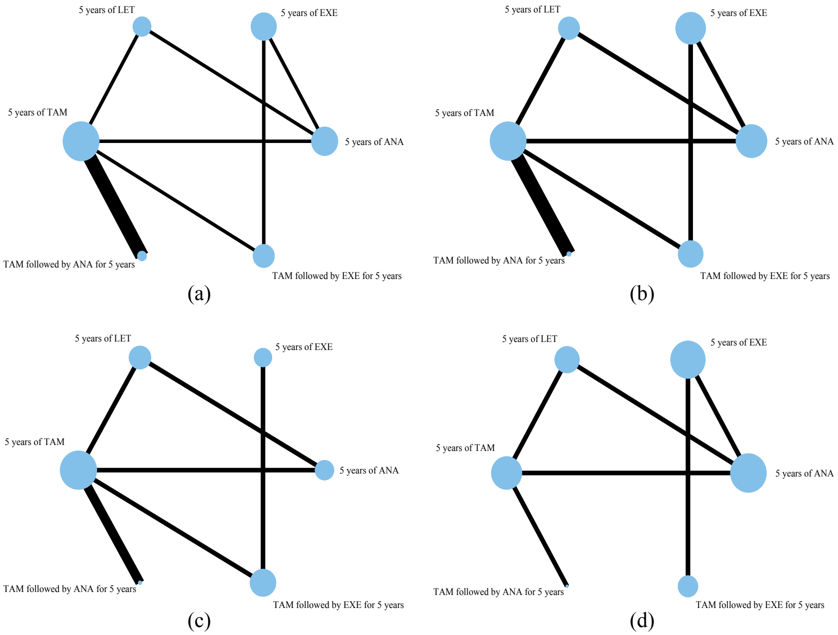


**Appendix 4.** Network structure diagrams of (a) bone fracture (b) cardiac events (c) thromboembolic events and (d) cerebrovascular events
**Abbreviations:** ANA, anastrozole; EXE, exemestane; LET, letrozole; TAM, tamoxifen.
