# Supplementary material for: Efficacy and Safety of Initial 5 Years of Adjuvant Endocrine Therapy in Postmenopausal Hormone Receptor-Positive Breast Cancer: A Systematic Review and Network Meta-Analysis
Source: Front Pharmacol. 2022 May 30;13:886954. doi: 10.3389/fphar.2022.886954 (PMC9198062; doi:10.3389/fphar.2022.886954)
Supplement: Supplementary file 4 [file Image2.PDF]

Appendix 2 Original data

Study	Treatment arms	DFS HR (95%CI)	OS HR (95%CI)	Bone fracture	Cardiac events	Thromboembolic events	Cerebrovascular events
GIM3-FATA 2018	(1) 5 years of AIs (2) 2 years of TAM followed by 3 years of AIs (ANA, EXE, LET)	0.89 (0.73-1.08)	0.72 (0.51-1.00)	NP	NP	NP	NP
FACE 2017	(1) 5 years of LET (2) 5 years of ANA	0.93 (0.80-1.07)	0.98 (0.82-1.17)	(1) 191/2049 (2) 166/2062	(1) 80/2049 (2) 46/2062	(1) 25/2049 (2) 24/2062	(1) 33/2049 (2) 30/2062
TEAM 2017	(1) 5 years of EXE (2) 2.5–3.0 years of TAM followed by EXE for a total of 5 years	0.96 (0.88–1.05)	0.98 (0.89-1.08)	(1) 249/4852 (2) 166/4814	(1) 391/4852 (2) 306/4814	(1) 94/4852 (2) 149/4814	(1) 87/4852 (2) 60/4814
N-SAS BC03 2014	(1) 1-4 years of TAM followed by ANA for a total of 5 years (2) 5 years of TAM	0.90 (0.65-1.24)	NP	(1) 5/347 (2) 9/349	(1) 2/347 (2) 3/349	(1) 1/347 (2) 0/349	NP
ITA 2013	(1) TAM followed by ANA for 5 years (2) 5 years of TAM	0.71 (0.52–0.97)	0.79 (0.52–1.21)	(1) 22/223 (2) 15/225	(1) 17/223 (2) 14/225	NP	NP
MA.27 2013	(1) 5 years of EXE (2) 5 years of ANA	1.02 (0.87-1.18)	0.93 (0.77-1.13)	(1) 358/3761 (2) 354/3759	(1) 38/3761 (2) 32/3759	NP	(1) 32/3761 (2) 38/3759

ABCSG-8 2012	(1) 2 years of TAM followed by 3 years of ANA<br>(2) 5 years of TAM	0.91 (0.75-1.10)	0.87 (0.65-1.16)	(1) 43/1865<br>(2) 28/1849	NP	NP	NP
ATAC 2010	(1) 5 years of ANA<br>(2) 5 years of TAM	0.86 (0.78–0.95)	0.95 (0.84-1.06)	(1) 183/3092<br>(2) 115/3094	(1) 76/3092<br>(2) 59/3094	(1) 64/3092<br>(2) 109/3094	(1) 31/3092<br>(2) 65/3094
BIG1-98 2009	(1) 5 years of LET<br>(2) 5 years of TAM	0.88 (0.78-0.99)	0.87 (0.75-1.02)	(1) 225/3975<br>(2) 159/3988	(1) 162/3975<br>(2) 153/3988	(1) 68/3975<br>(2) 154/3988	(1) 47/3975<br>(2) 49/3988
IES 2007	(1) 2-3 years of TAM followed by 2-3 years of EXE<br>(2) 5 years of TAM	0.76 (0.66-0.88)	0.83 (0.69-1.00)	(1) 100/2320<br>(2) 73/2338	(1) 382/2320<br>(2) 350/2338	(1) 28/2320<br>(2) 54/2338	NP
ARNO95 2007	(1) 2 years of TAM followed by 3 years of ANA<br>(2) 5 years of TAM	0.66 (0.44-1.00)	0.53 (0.28-0.99)	(1) 10/445<br>(2)10/452	(1) 9/445<br>(2) 4/452	(1) 0/445<br>(2) 6/452	(1) 3/445<br>(2) 1/452

Abbreviations: AI, aromatase inhibitor; EXE, exemestane; ANA, anastrozole; LET, letrozole; TAM, tamoxifen; DFS, disease-free survival; OS, overall survival; HR, hazard ratio; CI, confidence interval; NP, not reported.
