# Supplementary material for: Efficacy and Safety of Initial 5 Years of Adjuvant Endocrine Therapy in Postmenopausal Hormone Receptor-Positive Breast Cancer: A Systematic Review and Network Meta-Analysis
Source: Front Pharmacol. 2022 May 30;13:886954. doi: 10.3389/fphar.2022.886954 (PMC9198062; doi:10.3389/fphar.2022.886954)
Supplement: Supplementary file 5 [file Image3.PDF]

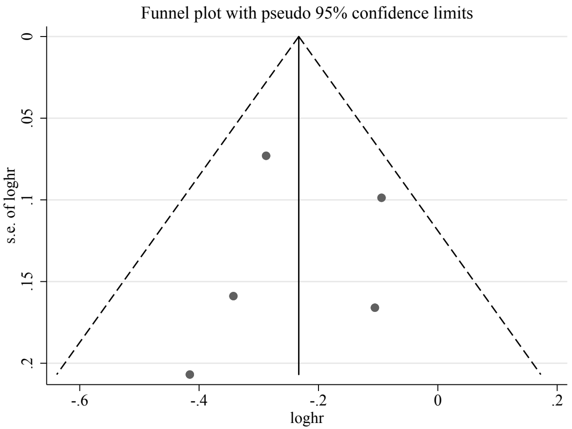

(a)

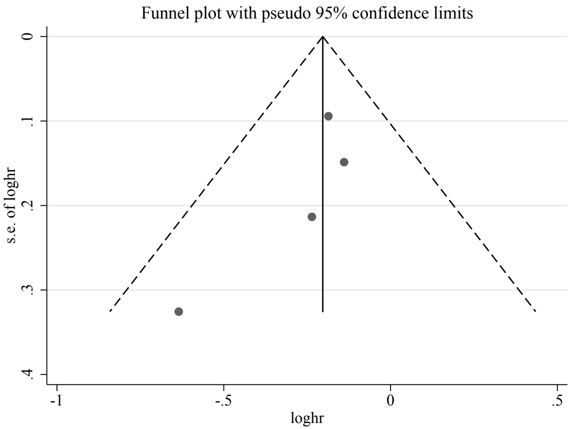

(b)

Appendix 3. Funnel plots of (a) DFS between TAM and TAM followed by an AI and (b) OS between TAM and TAM followed by an AI
Abbreviations: DFS, disease-free survival; OS, overall survival.
