# Supplementary material for: Efficacy and Safety of Initial 5 Years of Adjuvant Endocrine Therapy in Postmenopausal Hormone Receptor-Positive Breast Cancer: A Systematic Review and Network Meta-Analysis
Source: Front Pharmacol. 2022 May 30;13:886954. doi: 10.3389/fphar.2022.886954 (PMC9198062; doi:10.3389/fphar.2022.886954)
Supplement: Supplementary file 6 [file Image1.PDF]

(a) Main search algorithm:

(((((("Breast Neoplasms"[Mesh]) OR ((((((((((((((((((((((((((((((((((((Neoplasm, Breast) OR (Breast Tumors)) OR (Breast Tumor)) OR (Tumor, Breast)) OR (Tumors, Breast)) OR (Neoplasms, Breast)) OR (Breast Cancer)) OR (Cancer, Breast)) OR (Mammary Cancer)) OR (Cancer, Mammary)) OR (Cancers, Mammary)) OR (Mammary Cancers)) OR (Malignant Neoplasm of Breast)) OR (Breast Malignant Neoplasm)) OR (Breast Malignant Neoplasms)) OR (Malignant Tumor of Breast)) OR (Breast Malignant Tumor)) OR (Breast Malignant Tumors)) OR (Cancer of Breast)) OR (Cancer of the Breast)) OR (Mammary Carcinoma, Human)) OR (Carcinoma, Human Mammary)) OR (Carcinomas, Human Mammary)) OR (Human Mammary Carcinomas)) OR (Mammary Carcinomas, Human)) OR (Human Mammary Carcinoma)) OR (Mammary Neoplasms, Human)) OR (Human Mammary Neoplasm)) OR (Human Mammary Neoplasms)) OR (Neoplasm, Human Mammary)) OR (Neoplasms, Human Mammary)) OR (Mammary Neoplasm, Human)) OR (Breast Carcinoma)) OR (Breast Carcinomas)) OR (Carcinoma, Breast)) OR (Carcinomas, Breast))) AND ((primary OR early OR operable OR resectable OR curable OR non-metastatic OR non-advanced))) AND ((((((hormone receptor) OR (HR)) OR (ER)) OR (PR)) OR (("Receptors, Estrogen"[Mesh]) OR (((((((((((Estrogen Receptors) OR (Estrogen Receptor)) OR (Receptor, Estrogen)) OR (Receptors, Estrogen, Type II)) OR (Estrogen Receptor Type II)) OR (Estrogen Receptors Type II)) OR (Estrogen Nuclear Receptor)) OR (Nuclear Receptor, Estrogen)) OR (Receptor, Estrogen Nuclear)) OR (Receptors, Estrogen, Type I)) OR (Estrogen Receptor Type I)) OR (Estrogen Receptors Type I)))) OR (("Receptors, Progesterone"[Mesh]) OR (((((((Receptors, Progestin) OR (Progestin Receptors)) OR (Progesterone Receptor)) OR (Progesterone Receptors)) OR (Receptor, Progesterone)) OR (Progestin Receptor)) OR (Receptor, Progestin))))) AND ((positive) OR (+))) AND ((((((((((endocrine therapy) OR (adjuvant therapy)) OR (tamoxifen)) OR (anastrozole)) OR (letrozole)) OR (exemestane)) OR (aromatase inhibitor)) OR (AI)) OR (selective estrogen receptor modulator)) OR (SERM))) AND (randomized controlled trial[Publication Type])

(b) Sources:

2775 Total
1083 Pubmed
1250 Embase
442 Web of Science
1806 after duplications removed (969 duplications were removed)
72 after screening of titles and abstracts (1734 records were excluded for the following reasons: irrelevant studies, reviews, comments, case-reports, letters, conference abstracts, non-human studies, and other types of tumors)
11 after further evaluation (61 records excluded for the following reasons: non-RCT studies, n=33; non-English studies, n=7; patients in neoadjuvant or advanced settings were enrolled, n=13; lack of control or inappropriate control group, n=8)
11 included in network meta-analysis after full-text assessment for eligibility

Appendix 1. (a) Main search algorithm and (b) detailed sources of records
